# Psychometric properties of the Adolescent Resilience Questionnaire (ARQ) in a sample of Swedish adolescents

**DOI:** 10.1186/s12888-022-04099-4

**Published:** 2022-07-14

**Authors:** Doris Nilsson, Carl Göran Svedin, Frida Hall, Emelie Kazemi, Örjan Dahlström

**Affiliations:** 1 Department of Behavioural Sciences and Learning, Linköping University, Linköping, Sweden; 2 Department of Social Science, Marie Cederschiöld University, Stockholm, Sweden

**Keywords:** Resilience, Adolescents, ARQ, Psychometric

## Abstract

**Background:**

The importance of resilience, and interest in it, has increased markedly in recent years, based on the need to understand why some children and young people have a resilience to stress that others lack. At the same time, there has been a lack of instruments to measure resilience. The aim of this study was to translate the Adolescent Resilience Questionnaire (ARQ) into Swedish and investigate the psychometrics of this Swedish version.

**Methods:**

A normative sample of 616 students aged 15–17 was recruited through the school system in five different communities. Students filled out a digitalised composite form consisting of ARQ and three other standardised questionnaires, the Sense of Coherence Scale-13 (Soc-13), the Rosenberg Self-Esteem Scale (RSES) and the Relationship Questionnaire (RQ).

**Results:**

The ARQ, with five domains and twelve subscales, showed good alpha coefficients α = .95 for the total scale and subscales ranging between α = .70 to .91, except for the subscales Emotional insight (α = 0.69) and Empathy/Tolerance (α = .61). The convergent validity, which was tested for the first time in this study, was good, especially with the Internal Domain for both SOC-13 and RSES. The confirmatory factor analysis showed a satisfactory construct validity. Finally, some gender differences were seen, with boys scoring higher on the total ARQ scale.

**Conclusion:**

The study shows that the Swedish translation of ARQ has satisfactory psychometric properties. The ARQ could therefore be used as a tool for adolescents when evaluating the importance of resilience.

## Background

Adverse childhood experiences, ACEs, have been found to be common and have a great impact on health and psychosocial adaptation during childhood [1, 2], but also during the whole lifespan [3]. Traditionally, both in the general debate and within research, the focus has usually been on factors that contribute to why some children develop symptoms after an adverse childhood experience and much less on why some children seem to show resistance to developing symptoms and behavioural disorders.

Resilience has been defined as the ability to positively adapt or overcome adversity or stress [4, 5]. The American Psychological Association [6] defines resilience as a process whereby the individual adapts and recovers after adverse circumstances such as trauma, tragedy, threat, and/or high levels of stress. This approach gives the impression that resilience is something extraordinary, whereas it is in fact a part of the human being’s inherited biological capacity to adapt and cope with difficulties [5]. The complexity of resilience consists of far more than just the child’s strength to overcome life’s adversities. Nobody has immunity against hardship, and this way of viewing resilience is to ignore the possible influence of the surrounding environment [7]. Consequently, today’s research has increasingly taken into account the ecological theoretical models [8–10] and whether an individual’s ability to adapt to adversity and be resilient is influenced by the resources this person has in his/her surrounding environment, including biological, psychological, social, and socioeconomic factors in their milieu [11–13]. According to this approach, resilience factors or, more aptly, lack of resilience factors can also be risk factors; for example, in the way in which living in bad surroundings can be a risk factor to resilience in the face of traumatic experiences [14, 15]. So the ability to identify resilience factors using ecological models allows for the possibility to remedy areas identified as less developed [16, 17]. For example, which areas need to be better cared for: the individual, the family, the school, the community, or the wider society. Even though some people view resilience as fundamentally permanent and unchangeable, research has found that it can be improved by training and interventions [18].

Most of the research conducted on resilience has focused on resilience among adults. There are few studies among adolescents, and most instruments are constructed to measure resilience among adults [19]. In a review study covering the time-period 1989–2009 [20], 15 resilience measurement scales were studied; five of these instruments covered children and adolescents, but none of them scored high on the quality assessment. Similar results were found in a later review study of instruments measuring resilience among children and adolescents in the USA [21]. This must be seen as a shortcoming, given that adolescence can be viewed as a vulnerable stage in human development in relation to the risk of getting involved in health-risk behaviours and settings where stress is involved [22, 23]. To overcome the problems caused by the lack of knowledge concerning adolescence and the scarcity of studies taking the whole picture of resilience into account, Gartland and colleagues [24] developed a scale that included all the dimensions of the ecological model. They called the new scale The Adolescent Resilience Questionnaire (ARQ). The ARQ is a comprehensive tool and measures individual and environmental factors or resources encompassing culture, neighbourhood, school, and family, which makes it possible to favour implications in designing interventions [16, 17].

The ARQ comprises 88 items and 12 scales measuring resilience factors in the five domains: Self, Family, Peers, School, and Community [24]. According to Gartland et al. [24], the ARQ was developed based on the ecological transactional model, items were developed through an extensive literature review and focus-group discussions and underwent multiple cycles of psychometric testing and revision. The ARQ measures personal characteristics including Confidence, Emotional Insight, Negative Cognition, Social Skills, and Empathy & Tolerance, and relevant ecological domains include family, peers, school, and community [24].

The psychometrics, reliability, and construct validity of the ARQ, including confirmatory factor analyses, have been investigated in three recent studies and have been found robust and good in Spain [19], Iran [25] and Nepal [26]. Having an instrument with sound and robust psychometrics and investigated in the country and culture where it is to be used is important. However, the above-mentioned studies used only the ARQ in their investigations, except the study by Guliera et al. [19], in which one other instrument, the Youth Self Report (YSR), was correlated with some domains in the ARQ. Therefore, it is important to continue an investigation of the ARQ because it is an instrument that addresses factors from the whole theoretical ecological system and has several advantages.

Consequently, the aim of this study was to continue the investigation of Reliability and Validity of the ARQ in a sample of Swedish adolescents. To accomplish this, in addition to investigating the construct validity with confirmatory factor analysis, we developed some hypotheses, for the continuation of the establishment of validity. Based on the large body of research concerning resilience and sense of coherence [20, 27], resilience and self-esteem [28], and resilience and attachment styles [27, 29] the hypotheses were as follows:

Self-esteem measured by the Rosenberg Self-Esteem Scale (RSES) will correlate positively with the ARQ, and especially with the individual confidence domain measured by the ARQ.

2. Sense of coherence measured with the Sense of Coherence Scale-13 (Soc-13) will correlate positively with the total ARQ and the underlying domains, such as the Individual domain and its subscales of Confidence, Emotional Insight, Negative Cognition, Social Skills, and Empathy/Tolerance on the ARQ.

3. Secure attachment styles measured with the Relationship Questionnaire (RQ) will correlate positively with the ARQ and insecure styles will correlate negatively.

## Method

### Participants

The participant adolescents were recruited through schools in five different communities in the middle of Sweden located, due to convenience, within commuting distance of the university. Fifty-four schools were approached for participation and eight agreed to participate constituting 34 different classes covering different socioeconomic groups and study programs (theoretical and vocational).

In this study, 650 adolescents aged 15–17 years were asked to participate. Out of this sample of 650 adolescents, 22 were excluded because they had passed 18 years of age or had given nonsense answers, and 12 did not complete the questionnaire, rendering a study group of 616 adolescents, a participation rate of 94.8%. There were 295 girls and 319 boys, while two answered ‘other’ on the question of gender. The mean age was 16.4 years (SD = 0.50), 70 adolescents were born in another country (11.4%), of whom 47 were born in a country outside Europe (7.6%) and 23 (3.7%) in another European country.

Data was collected in Spring 2020, during February and March. The study was designed as a community/school based, cross sectional study to investigate the psychometric qualities of ARQ.

### Procedure

A letter was sent out by email to the headmaster/mistress of each of the schools, followed by telephone contact one or two weeks later. When a school decided to participate, a letter with information about the research was sent out. The headmaster/mistress who had said yes gave contact possibilities to the teacher of the class and a date for the research was decided.

Before all survey occasions, teachers and mentors in all classes were given oral information about the study, and information sheets were issued about where students can apply for support if needed. At all data collection sessions, at least one of the authors was present throughout the session (F.H. or E.K.) and was available to answer questions and respond to any reactions. Before the students' participation in the study, an oral review was held about the purpose of the study and information about the study with emphasis on voluntariness, anonymity, and instructions for implementation. After the oral review, information letters and consent forms were distributed to all students. After signing consent forms, the students were referred to the questionnaire survey via the website www.iterapi.se., which is connected to the platform at Linköping University and is considered completely safe and secure. Since students in Sweden use laptops, both during lessons and for homework, the online solution was decided on together with the headmasters of the schools. It took less than a lesson (60 min) to complete the questionnaire.

The questionnaire package consisted firstly of a page covering demographic data and then the measures.

### Questionnaires

#### Adolescent Resilience Questionnaire (ARQ)

The ARQ is a self-report instrument intended to identify the adolescent’s ability to be resilient even when he/she is experiencing difficult circumstances [24]. It was developed by Gartland and colleagues [24] to investigate internal and social resilience factors in adolescents aged 11–19 years during the previous six months. The instrument has 88 items, with 12 inherent scales divided into five domains. The domains are: *a*) Internal: with subscales self-esteem, emotional insight, negative cognition, social skills, and empathy/tolerance, b) Family: with subscales connectedness and availability, c) Peers: with subscales connectedness and availability, d) School: with subscales supportive environment and connectedness, and e) Community.

The items are formulated into claims such as: “My family listens to me”, or “My life has a sense of meaning to me”. It covers the previous six months and the answer options lie on a five-point Likert scale, ranging from 1 (all the time) to 5 (never). In the Australian study [24], 451 students from 11 schools answered the formula and the authors showed it to have good internal consistency, with a Cronbach’s alpha ranging between 0.70 and 0.90 for all scales except the subscale *Friends* – availability.

The ARQ was translated into Swedish by three researchers with great knowledge of the subject and with permission from D. Gartland [30]. When consensus was reached by the researchers, the ARQ was back-translated by a native English professor. The necessary corrections were then made until a final version was developed.

### Sense of coherence scale-13 (Soc-13)

A Swedish-translated version of the Sense of Coherence Scale (SOC), created by Antonovsky [31], was used in the present study. This is a well-known scale and has been used in many studies. The questionnaire is based on Antonovsky’s early theoretical model, which aimed to increase understanding of the relationship between coping strategies, stress, and health. SOC is understood in terms of three components: comprehensibility, manageability, and meaningfulness [31]. In the present study, the SOC-13 was used. SOC-13 is a shorter version of SOC-29 [32]. The questions relate to different areas of life and are answered on a seven-point Likert scale, ranging from 1 (very seldom or never) to 7 (very often). A high overall score suggests a strong SOC. It has been shown that SOC-13 has good validity and reliability and is valid as a cross-cultural instrument [32], and validated in Sweden [33]. In this study, *Cronbach’s alpha* was found to be 0.86.

### Rosenberg self-esteem

The Rosenberg Self-Esteem Scale [34] is a scale designed to measure the concept of global self-esteem. This concept is understood in its common definition as a person’s overall sense of worth [35]. Individuals report how true the ten statements are for them. Four answering options are used, ranging from 0 (strongly disagree) to 3 (strongly agree). The total score ranges from 0 to 30, with high values indicating high self-esteem. In a study where 16,988 participants in 53 countries answered RSES, the total average alpha level for all countries, was 0.81, which indicates good internal consistency [36]. The scale has been validated in Sweden [37] In this study, *Cronbach’s alpha* was found to be 0.91.

### Relationship questionnaire (RQ)

RQ is a self-report instrument which has four items, intended to indicate participants’ attachment style [38]. The four attachment styles identified in the RQ are thought to measure how an individual looks upon and behaves in his/her relationships. They are believed to be an effect of the person’s relational history. The four attachment styles are: *secure, dismissing, preoccupied, and fearful*, where the last three indicate an insecure attachment style [38]. On the questionnaire, the participant marks on a seven-point scale ranging from 1 (disagree strongly) to 7 (agree strongly) how much they recognise themselves in the description of each attachment style. Question number 2 is designed to identify a secure attachment style and questions 1, 3, and 4 indicate insecure styles. The scale has been translated into Swedish [39] and was validated in 2001 [40] on a sample of adults.

### Ethical considerations

This study was approved by the This study was approved by the regional ethical review board at Linköping University (Ref. no. 220–08) and the authors have followed the ethical codex concerning information, consent, and usefulness (Swedish Research Council, 2002). There were no agreements, rewards, or payments to participate.

### Statistical analyses

Internal consistency for the ARQ domains were examined using Cronbach’s alpha. To test for construct validity, the Gartland [24] model was fitted with the study sample using confirmatory factor analysis (CFA) with the mean- and variance-adjusted weighted least squares (WLSMV) estimator [41]. Adjustment of the model to Swedish adolescents was made by excluding items not loading on their respective factor. Model fit was examined by Overall model fit and was tested by: Root Mean Square Error of Approximation (RMSEA), Comparative Fit Index (CFI), Tucker-Lewis Index (TLI) and Standardised Root Mean Square Residual (SRMR). Using guiding principles set out by Brown [42] and Schreiber et al. [43], several different fit indices were used. For convenience of reporting, a model was judged as having good fit when the overall picture of fit indices indicated good fit, and excellent if all of them indicated good fit: (χ^2^/df < 3, RMSEA ≤ 0.05, CFI and TLI ≥ 0.95, and SRMR < 0.08 [43–45].

Concurrent validity was examined by means of correlations between factor scores generated from CFA and a) SOC-13, and b) RSES, using Kendall’s tau. Notably, Kendall’s tau values are generally 66–75% of the size of Pearson correlations [46], and for comparative purposes the more conservative 75% was used, i.e. Pearson correlations of 0.10, 0.30, and 0.50 (often considered as small, moderate, and large) are comparable with Kendall’s *τ* values of 0.075, 0.225 and 0.375.

Factor analyses were performed using Mplus version 8.4 [47], while other analyses were performed using RStudio [48] with R version 4.0.3 [49].

## Results

### Descriptives

Descriptive statistics for different scales are shown in Table 1. Girls showed significantly lower scores for ARQ total, t(604.97) = 4.51, p < 0.001, *r* = 0.18, and significantly lower scores for both SOC-13 scores, t(605.27) = 6.88, p < 0.001, *r* = 0.27, and RSES scores, t(603.28) = 5.63, p < 0.001, *r* = 0.22. There were also significantly higher proportions of boys who associated themselves with a secure attachment style and consequently a lower insecure attachment style compared with the girls, χ^2^(1, N = 614), 5.26, *p* = 0.02, Φ = 0.09. There were no significant differences in ARQ, SOC-13, RSES, or proportions of secure attachment style between adolescents born in different geographical regions (see Table 1).

**Table 1:** Descriptive data for the used questionnaires

	**All**	**Gender**			**Born**		
		Female	Male	Other	Sweden	Europe	Outside Europe
*n* (%)	616 (100%)	295 (47.9%)	319 (51.8%)	2 (0.3%)	546 (88.6%)	23 (3.7%)	47 (7.6%)
ARQ^a^ *M (SD)*	309.16 (38.59)	301.91 (38.53)	315.75 (37.41)***	328.50 (65.76)	309.98 (38.43)	312.13 (41.84)	298.26 (37.93)
SOC-13^b^ *M (SD)*	55.62 (13.40)	51.86 (13.11)	59.06 (12.76)***	61.00 (9.90)	55.71 (13.50)	57.7 (14.28)	53.57 (11.75)
RSES^c^ *M (SD)*	33.44 (9.89)	31.23 (9.45)	35.44 (9.06)***	39.00 (14.14)	33.29 (9.44)	34.35 (9.54)	34.70 (10.07)
RQ^d^			*				
Secure *n* (%)	209 (33.9%)	86 (29.1%)	122 (38.2%)	1 (50.0%)	186 (34.1%)	8 (34.8%)	15 (31.9%)
Unsecure *n* (%)	407 (66.1%)	209 (70.9%)	197 (61.8%)	1 (50.0%)	360 (65.9%)	15 (65.2%)	32 (68.1)

^a^Adolescent Resilience Questionnaire, total score

^b^ Sense of Coherence Scale-13, total score

^c^ Rosenberg Self-esteem Questionnaire, total score (corrected for one item missing in the survey)

^d^ Relationship Questionnaire, secure and unsecure attachment styles

Difference between female/male *** *p* < .001, ** *p* < .01, * *p* < .05

Difference between regions all non-significant

### Reliability

Internal consistency measured with Cronbach’s alpha ranged between 0.70–0.91 in all domains and subscales, except for the subscales internal empathy/tolerance and emotional insights, which were found to be 0.61 and 0.69, respectively. For the total ARQ scale, Cronbach’s alpha was 0.95 (see Table 2).

**Table 2:** Cronbach’s alpha for the scales of Adolescent Resilience Questionnaire (ARQ) with questions verified by confirmatory factor analysis

**Domains** and *Factors*	**Cronbach’s alpha**	**n items**
**Internal domain**	.91	40
* Self confidence*	.83	8
* Negative cognition*	.85	8
* Empathy/Tolerance*	.61	8
* Emotional insight*	.69	8
* Social skills*	.80	8
**Family domain**	.90	11
* Connectedness*	.88	8
* Availability*	.78	3
**Peers domain**	.88	15
* Connectedness*	.82	7
* Availability*	.80	8
**School domain**	.84	16
* Supportive environment*	.82	8
* Connectedness*	.70	8
**Community domain**	.86	6
* Connectedness*	.86	6
**ARQ-total**	.95	88

### Validity

#### Construct validity

##### Factor analysis

A CFA testing of how well the Gartland et al. [24] model fits with the Swedish sample of adolescents indicated poor fit (*p* = 0.83) for item 28 (“I expect people to live up to my standards”). Therefore, item 28 was excluded, and a new CFA was performed which showed acceptable fit with significant factor loadings on all items (χ^2^/df = 2.4, RMSEA = 0.048, 90% CI: 0.047–0.049, CFI = 0.86, TLI = 0.86, SRMR = 0.071) (see Table 3).

**Table 3:** Confirmatory factor analysis testing construct validity of the Gartland model (Gartland et al., 2011) but where item ARQ28 has been removed.

**Domains,** *Factors* and Items	Question	Estimate (Std Err)	p
**Individual domain**			
*Confidence*			
13	I feel hopeful about my life	1	
1	My life has a sense of purpose	0.850 (0.035)	< .001
6	I feel good about myself	0.945 (0.029)	< .001
7	If I have a problem I can work it out	0.834 (0.036)	< .001
18	I am confident that I can achieve what I set out to do	0.833 (0.032)	< .001
19	I am a person who can go with the flow	0.353 (0.046)	< .001
22	I feel confident that I can handle whatever comes my way	0.902 (0.033)	< .001
39	I feel confident to do things by myself	0.621 (0.045)	< .001
*Negative cognition*			
20 ^r^	I can´t stop worrying about my problems	1	
2^r^	I worry about the future	0.752 (0.039)	< .001
5 ^r^	My feelings are out of my control	0.818 (0.043)	< .001
8 ^r^	I dwell on the bad things that happen	0.758 (0.044)	< .001
11 ^r^	I tend to think the worst is going to happen	0.860 (0.036)	< .001
30 ^r^	When things go wrong, I tend to give myself a hard time	0.811 (0.042)	< .001
32 ^r^	I just can´t let go of bad feelings	0.923 (0.038)	< .001
38 ^r^	If something upsets me it affects how I feel about everything	0.627 (0.045)	< .001
*Empathy/tolerance*			
23	I am able to let go of things I can’t control	1	
3 ^r^	I am easily frustrated with people	0.843 (0.084)	< .001
9	I am patient with people who can´t do things as well as I can	0.418 (0.079)	< .001
17 ^r^	I get frustrated when people make mistakes	0.417 (0.079)	< .001
25 ^r^	I push myself too hard to do what everyone else do	0.964 (0.083)	< .001
35	I think about other people’s feelings before I say things	0.256 (0.083)	.002
37	Other people’s feelings are easy for me to understand	0.470 (0.083)	< .001
*Emotional insight*			
16	If I cant´t handle something I find Help	1	
4	I take it easy on myself when I am not feeling well	0.772 (0.054)	< .001
10	I look for what I can learn out of bad things that happen	0.562 (0.058)	< .001
14	When I am feeling down, I take extra special care of myself	0.923 (0.051)	< .001
26	I can change my feelings by changing the way I see things	0.403 (0.061)	< .001
27	I try to find meaning in the things that happen to me	0.329 (0.061)	< .001
36	If I have a problem, I know there is someone I can talk to	0.997 (0.050)	< .001
40	I think things through carefully before making decisions	0.378 (0.062)	< .001
*Social skills*			
29	I find it easy talking to people my age	1	
12 ^r^	I feel helpless when faced with a problem	0.965 (0.056)	< .001
15	I can express my opinions when I am in a group	0.944 (0.052)	< .001
21 ^r^	I find it hard to express myself to others	0.970 (0.051)	< .001
24 ^r^	I have trouble explaining how I am feeling	0.929 (0.053)	< .001
31 ^r^	I am a shy person	0.897 (0.053)	< .001
33	I can share my personal thoughts with others	0.913 (0.050)	< .001
34 ^r^	I find it hard to make important decisions	0.729 (0.060)	< .001
**Family domain**			
*Connectedness*			
45	My family listens to me	1	
41	I do fun things with my family	0.913 (0.025)	< .001
42	I get to spend enough time with my family	0.743 (0.033)	< .001
43	My family understands my needs	0.987 (0.022)	< .001
44	We do things together as a family	0.938 (0.024)	< .001
46 ^r^	People in my family expect too much of me	0.294 (0.048)	< .001
48	I enjoy spending time with my family	0.854 (0.028)	< .001
49	My family helps me to believe in myself and my abilities	0.983 (0.020)	< .001
*Availability*			
51	I I have a problem there is someone in my family I can talk to	1	
47	There is someone in my family that I feel particularly close to	0.530 (0.043)	< .001
50	There is someone in my family I can talk about anything	0.863 (0.026)	< .001
**Peers domain**			
*Connectedness*			
66	I feel confident around people my age	1	
52	When I am down I have friends that help cheer me up	0.982 (0.049)	< .001
54	I have a group of friends that I keep in touch with regularly	0.903 (0.052)	< .001
57	I have friends who make me laugh	0.989 (0.056)	< .001
61	I get to spend enough time with my friends	0.857 (0.054)	< .001
63	I enjoy being around people my age	0.859 (0.045)	< .001
65	I have a friend I can trust with my private thoughts and feelings	0.768 (0.053)	< .001
*Availability*			
53 ^r^	I find it hard making friends	1	
55	Making new friends is easy	0.959 (0.026)	< .001
56 ^r^	I feel left out of things	0.744 (0.034)	< .001
58	I am happy with my friendship group	0.808 (0.041)	< .001
59 ^r^	I find it hard to stay friends with people	0.719 (0.037)	< .001
60 ^r^	I prefer to do things on my own	0.334 (0.047)	< .001
62 ^r^	I wish I had more friends I felt close to	0.629 (0.038)	< .001
64 ^r^	I feel shy around people my age	0.827 (0.031)	< .001
**School domain**			
*Supportive environment*			
67	My teachers are caring and supportive of me	1	
68	I have a teacher that I feel looks out for me	0.756 (0.049)	< .001
71	My teachers provide me with extra helo if I need it	0.866 (0.044)	< .001
73	There is an adult at school I could talk to if I had a personal problem	0.870 (0.051)	< .001
77	I get involved with school activities	0.769 (0.051)	< .001
78	I feel that what I say counts at school	0.997 (0.051)	< .001
79	At school students help to decide and plan things like school activities and events	0.646 (0.052)	< .001
81	My teachers notice when I am doing a good job and let me know	0.757 (0.054)	< .001
*Connectedness*			
76	I enjoy going to school	1	
69 ^r^	I hate going to school	0.981 (0.034)	< .001
70	I try hard in school	0.441 (0.054)	< .001
72	I join class discussions	0.917 (0.041)	< .001
74 ^r^	My teachers expect too much of me	0.164 (0.059)	.005
75	I participate in class	0.867 (0.042)	< .001
80 ^r^	I am bored at school	0.631 (0.046)	< .001
82	Getting good marks is important to me	0.317 (0.056)	< .001
**Community domain**			
*Connectedness*			
83	I trust people in my neighbourhood	1	
84	I like my neighbourhood	0.933 (0.032)	< .001
85	There is an adult in my neighbourhood I could talk to about a problem	0.802 (0.039)	< .001
86	People in my neighbourhood are caring	0.993 (0.027)	< .001
87	People in my neighbouhood treat other people fairly	0.765 (0.041)	< .001
88	The people in my neighbourhood look out for my	0.891 (0.033)	< .001

^r^Reveresed items, values are reversed before included in analysis

##### Correlations between subscales

The intercorrelations between the 12 subscales were all significant, with all but eight considered moderate to large. The correlations between all the subscales are presented in Table 4.

**Table 4:** Correlations (Kendall’s τ*) between the Different domains on the ARQ

	IntSelf	IntNegCo	IntEmpTol	IntEmoIns	IntSoc	FamCon	FamAva	PeersCon	PeersAva	SchoolSup	SchoolCon
^a^IntSelf											
^b^IntNegCog	0.58^***^										
^c^IntEmpTol	0.57^***^	0.69^***^									
^d^IntEmoIns	0.70^***^	0.50^***^	0.58^***^								
^e^IntSoc	0.64^***^	0.53^***^	0.46^***^	0.53^***^							
^f^FamCon	0.43^***^	0.29^***^	0.29^***^	0.48^***^	0.33^***^						
^g^FamAva	0.38^***^	0.25^***^	0.25^***^	0.51^***^	0.28^***^	0.66^***^					
^h^PeersCon	0.31^***^	0.18^***^	0.21^***^	0.31^***^	0.48^***^	0.28^***^	0.25^***^				
^i^PeersAva	0.39^***^	0.35^***^	0.31^***^	0.31^***^	0.68^***^	0.26^***^	0.21^***^	0.68^***^			
^j^SchoolSup	0.37^***^	0.24^***^	0.32^***^	0.47^***^	0.31^***^	0.36^***^	0.32^***^	0.29^***^	0.21^***^		
^k^SchoolCon	0.38^***^	0.30^***^	0.32^***^	0.38^***^	0.39^***^	0.30^***^	0.23^***^	0.27^***^	0.28^***^	0.52^***^	
^l^ComCon	0.24^***^	0.17^***^	0.14^***^	0.25^***^	0.22^***^	0.34^***^	0.25^***^	0.23^***^	0.19^***^	0.27^***^	0.19^***^

^a^ Confidence (self and future), ^b^ Negative cognition,^c^ Emotional tolerance, ^d^ Emotional insight, ^e^ Social skills, ^f^ Family connectedness, ^g^ Family aviliability, ^h^ Peers connectedness, ^I^ Peers availability, ^j^ School supportive,^k^ School connectedness, ^l^ Community connectedness

^*^Kendall’s τ values of .075, .225 and .375 are considered small, moderate, and large

**Table 5:** Correlations (Kendall’s τ*) between ARQ and Sense of Coherence (SOC-13) and between ARQ and Rosenberg scores (RSES)

**Domains** and *Factors*	**SOC-13**	**RSES**^**a**^
**Internal domain**	.59	.59
*Self confidence*	.51	.60
*Negative cognition*	.57	.46
*Empathy/Tolerance*	.29	.25
*Emotional insight*	.38	.43
*Social skills*	.39	.43
**Family domain**	.36	.33
*Connectedness*	.38	.34
*Availability*	.24	.23
**Peers domain**	.27	.29
*Connectedness*	.19	.22
*Availability*	.30	.30
**School domain**	.30	.30
*Supportive environment*	.25	.24
*Connectedness*	.28	.29
**Community domain**		
*Connectedness*	.18	.16
**ARQ-total**	.55	.53

*All correlations are significant with p < .001. Kendall’s tau, with values of .075, .225 and .375 are considered small, moderate and large.

^a^ RSES scores were corrected for one item missing in the survey

##### Convergent validity

The correlations between the ARQ internal domains and SOC-13 varied between Kendall’s tau 0.46 and 0.61, which are all significant and considered strong (see Table 5). The correlations between the ARQ and RSES varied between Kendall’s tau 0.50 and 0.61, also all significant and considered strong. The correlations between other domains and their subscales were lower, but all significant at the same level (p < 0.001).

The ARQ total scale also correlated significantly with the total scales of SOC-13 and RSES, at 0.54 and 0.53, respectively. The ARQ total scale also correlated with RQ secure (τ = 0.28, *p* < 0.001), preoccupied, (τ =—0.19, *p* < 0.001), and fearful (τ = -0.30, *p* < 0.001) attachment styles, but not with the dismissive attachment style (τ = -0.01, *p* = 0.82) (data not shown in Table 5).

## Discussion

The purpose of this study was to investigate the psychometric properties of the ARQ, a resilience questionnaire for adolescents developed by Gartland and colleagues [24]. The scale has several dimensions which take the ecological theoretical models into account.

The objective was to investigate the psychometrics of the ARQ and to continue the establishment of its validity. The psychometrics, including reliability, construct, and convergent validity of the ARQ were found to be sound and all our hypotheses were confirmed. The results can be summarised in five main findings.

Firstly, this was the first study in Sweden using the translated version of the Adolescent Resilience Questionnaire, and even though the questionnaire is lengthy, with many questions, our experience was that it is easy to administer and seems to be readily comprehended by the students in the studied age group. This increases the usefulness of the ARQ for future studies.

Secondly, reliability in this study must be considered excellent, with an alpha of 0.95 for the total scale and alpha above 0.80 on eight subscales, with two between 0.70 and 0.78. However, internal empathy/tolerance and emotional insight had less good alpha, with only 0.61 and 0.69 respectively. The results of this study were in line with what has been found in other studies, which have found alpha values ranging between *α* = 0.64–0.88 [24], *α* = 0.70–0.79 [25] and α = 0.64–0.86 [26]. Guilera and colleagues [19], in their sample, found alpha values ranging between α = 0.78–0.82, except for the subscales emotional insight, where they found an α = 0.60, and empathy/tolerance, for which α = 0.38. In summary, in three previous studies, and also in this study, for the subscale empathy/tolerance it seems to be difficult to get more than a low limit for the internal consistency. However, this was not the case in the study by Cheragi et al. [25].

Thirdly, in order to study the construct validity, the confirmatory factor analysis showed a fairly good model fit (three out of five fit-indices indicated good model fit and the remaining two – TLI and CFI – both mainly indicating similar type of fit; 43) were close to the cut-offs specified), something which has also been found in studies from Spain [19], Iran [25], and Nepal [26]. However, item number 28 was removed because the formulation of that item did not fit, and it could be understood from the Swedish language that this item did not have the same meaning in Sweden. All items had loadings that were significant. It should be noted that item 28 belongs to the subscale empathy/tolerance where several studies, including this one, had alphas at the lower limit.

Fourthly, resilience according to the construct of the ARQ scale is based on the theories of the ecological model [8–10]. This includes the domains of the Individual, Family, Peers, School, and Community. These five domains also have subscales intended to identify factors such as self-confidence, emotional insight, negative cognition, social skills, empathy, and tolerance (Individual domain), connectedness and availability (Family and Peers domains), a supportive environment and connectedness (School domain), and finally connectedness (Community domain). As a construct, these domains with their subscales should be interconnected to some degree, but not overlapping. The results showed significant intercorrelations between subscales, varying mostly between large to moderate, while the Community domain had some small but statistically significant intercorrelations. The largest correlations were shown among the subscales within the same domain, except for the Internal: social skills and Peers’ availability, which showed a slightly higher correlation, and for the Internal: social skills and Internal empathy/tolerance, which showed a slightly smaller correlation. This result must be seen as strengthening the ecological theory behind the ARQ.

Fifthly, the convergent validity was measured by correlation of the sense of coherence, SOC-13, and Rosenberg’s Self-Esteem Scale with the ARQ domains and total scale. It was found to be large with the internal domain, but also large with the total scale. High Resilience has been found in other studies to correlate positively with Rosenberg’s Self-Esteem Scale and the SOC [50]. So, it can be argued that sense of coherence and self-esteem are positively interconnected with resilience measured using the ARQ, which was hypothesised and strengthens the validity of the ARQ. Also, the hypothesis concerning the Relationship Questionnaire (RQ) was shown to be confirmed because a secure attachment style showed a significant positive correlation with the ARQ, and the more insecure attachment styles were, as expected, negatively associated with resilience. The interpretation of this suggests that attachment style is associated with resilience as measured with the ARQ. Similar findings have been found in a study by Bender and Ingram [29], who also found that secure attachment style related to resilience. However, this was true in such a way that self-efficacy and selfcare partially mediated the relationship between attachment and resilience, a pattern of relationship that was found between all the dimensions of attachment [28, 29].

Overall, the results of this study have shown that the psychometrics of the ARQ are sound and good, although there may be room for alternative models with further improvements. The results can also be seen to strengthen the theory behind the scale, the model of the ecological theories developed by Bronfenbrenner [8, 9] and Belsky [10]. The questionnaire is rather long, but if it is to have the ability to cover all the domains involved in the ecological theory system model this is perhaps necessary since resilience is a complex concept and must be seen in the context of the whole system complex. It should be noted that, in this study, it was shown that girls scored significantly lower on the ARQ, which also has been found in a previous study in Nepal [51], but also on the SOC-13, and Rosenberg’s Self-Esteem Scale, and also scored lower on the secure attachment style. Although beyond the scope of this paper, this is something that ought to be investigated in future studies, including trauma experiences, since we know from earlier studies that interpersonal traumas are more frequent among girls [1] and that there are clear associations between both psychological distress and lowered self-esteem [52] and traumatic experiences. We can see two more future areas to study scientifically. The first is whether it is possible to reduce the number of questions in the questionnaire while at the same time retaining the psychometric qualities. This research has recently begun by evaluating which items can be included in a shorter version of ARQ. The second is to use the questionnaire as a basis to evaluate whether high resilience can reduce the consequences of various traumatic events.

### Strengths and limitations

A strength of this study is the large sample size and the high response rate, together with almost equal proportions of male and female participants. Even though eight out of 54 schools responded, the responding the catchment area of the responding schools is fairly representative of Sweden as a whole concerning socioeconomical data [53], indicating minimal between-class bias. According to Fincham [54], a response rate of at least 80% is required to achieve representativeness for a study population, and even though the sample did not cover 80% of all possible adolescents at all invited schools, a response rate of 94.8% among invited adolescents indicates minimal within-class bias. Therefore, even though some cautiousness should be taken, we consider the findings rather generalizable to Swedish adolescents. A limitation, or a strength, when digitalising the questionnaire package can be discussed [55]. However, it has been found to fulfil the purpose [56]. One argument for the strength is that young people in Swedish schools are using the internet very frequently, both in education and during their leisure time.

One limitation is that there is no test–retest reliability, which could further establish the psychometrics. Another possible limitation is the use of Cronbach’s alpha as measure for reliability. We did, however, calculate alternative measures [57] which, if anything, showed stronger reliability. To be coherent with other validations of ARQ [19, 24–26] we chose to present the more conservative (in this case) Cronbach’s alpha.

## Conclusion

The study has shown that the Swedish version of the translation of the ARQ has satisfactory psychometric properties. The validity of the ARQ has been strengthened and so also the ecological theory behind the scale. The ARQ could therefore be used as a tool for adolescents when evaluating the importance of resilience.

## Data Availability

Data can be found at Linköping University in contact with first and last authors. All questionnaires in this study have already been published elsewhere and can be found: *Adolescent Resilience Questionnaire (ARQ)* Original English version is available in supplementary material in reference 24 in the manuscript: Gartland, D., Bond, L., Olsson, C. A., Buzwell, S., & Sawyer, S. M. (2011). Development of a multi-dimensional measure of resilience in adolescents: The adolescent resilience questionnaire. *Medical Research Methodology, 11*(134), 1–10, https://doi.org/10.1186/1471-2288-11-134. *Sense of Coherence Scale-13 (Soc-13)* An English version of SOC-13 is a subset of SOC-29. It is for example available in Table 2 in Sardu et al., (2012). Antonovsky’s Sense of Coherence Scale: Cultural Validation of Soc Questionnaire and Socio-Demographic Patterns in an Italian Population, Clinical Practice & Epidemiology in Mental Health, 2012, 8, 1–6. https://clinical-practice-and-epidemiology-in-mental-health.com/contents/volumes/V8/CPEMH-8-1/CPEMH-8-1.pdf *Rosenberg Self-Esteem* The scale is referred to in references 30 and 31 in the manuscript, but for convenience the English questions can be found directly here https://fetzer.org/sites/default/files/images/stories/pdf/selfmeasures/Self_Measures_for_Self-Esteem_ROSENBERG_SELF-ESTEEM.pdf *Relationship Questionnaire (RQ)*An English version is available in Appendix B in reference 33 in the manuscript: Bartholomew, K. & Horowitz, L. M. (1991). Attachment styles among young adults: A test of a four-category model. Journal of Personality and Social Psychology, 61, 226–244. https://doi.org/10.1037/0022-3514.61.2.226

## Abbreviations

ARQ: Adolescent Resilience Questionnaire
ACE: Adverse Childhood Experiences
CFI: Confirmatory Factor Analysis
CFI: Comarative Fit Index
RQ: Relationship Questionnaire
RSMA: Root Mean Square Error Approximation
SOC 13: Sense of Coherence -thirteen
SRMR: Standardised Root Mean Square Residual
YSR: Youth Self Report
